# A pan-cancer analysis of the oncogenic and immunological roles of apolipoprotein F (APOF) in human cancer

**DOI:** 10.1186/s40001-023-01156-w

**Published:** 2023-06-14

**Authors:** Xu Shi, Dechao Feng, Dengxiong Li, Ping Han, Lu Yang, Wuran Wei

**Affiliations:** Department of Urology, Institute of Urology, West China Hospital, Sichuan University, Guoxue Xiang #37, Chengdu, 610041 China

**Keywords:** Apolipoprotein F, Pan-cancer, Prognosis, Breast invasive carcinoma, Kidney chromophobe, Liver hepatocellular carcinoma, Prostate adenocarcinoma

## Abstract

**Background:**

Apolipoprotein F (APOF) has been less studied in cancers. Thus, we aimed to perform a pan-cancer analysis of the oncogenic and immunological effects of APOF on human cancer.

**Methods:**

A standardized TCGA pan-cancer dataset was downloaded. Differential expression, clinical prognosis, genetic mutations, immune infiltration, epigenetic modifications, tumor stemness and heterogeneity were analyzed. We conducted all analyses through software R (version 3.6.3) and its suitable packages.

**Results:**

Overall, we found that the common cancers differentially expressed between tumor and normal samples and prognostic-associated were BRCA, PRAD, KIRP, and LIHC in terms of overall survival (OS), disease-free survival (DFS) and progression-free survival (PFS). The pan-cancer Spearman analysis showed that the mRNA expression of APOF was negatively correlated with four tumor stemness indexes (DMPss, DNAss, ENHss, and EREG-METHss) with statistical significance for PRAD and was positively correlated for LIHC. In terms of BRCA and PRAD patients, we found negative correlation of APOF with TMB, MSI, neo, HRD and LOH. The mutation frequencies of BRCA and LIHC were 0.3%. APOF expression was negatively correlated with immune infiltration and positively correlated with tumor purity for PRAD patients. The mRNA expression of APOF was negatively associated with most TILs for LIHC, B cells, CD4+ T cells, neutrophils, macrophages and dendritic cells, but was positively associated with CD8+ T cells.

**Conclusions:**

Our pan-cancer study offered a relatively comprehensive understanding of the roles of APOF on BRCA, PRAD, KIRP, and LIHC.

**Supplementary Information:**

The online version contains supplementary material available at 10.1186/s40001-023-01156-w.

## Introduction

The apolipoprotein F (APOF) gene is located at 12q13.3, and its product was identified as a minor apolipoprotein in plasma first in 1978, which may be involved in cholesterol (CE) transport and/or esterification [1]. APOF was found to be expressed at a considerable higher level in normal liver than in other parts of human organs, mainly participating in lipoprotein metabolism [2, 3]. APOF inhibits cholesteryl ester transfer protein (CETP) activity, among which it preferentially inhibits transfer events involving low-density lipoprotein (LDL) [4]. In this way, APOF can reduce LDL levels and enhance reverse CE transport in mouse model [5]. Conversely, APOF knockdown enhances the transfer of cholesteryl esters to LDL [6]. Meanwhile, APOF overexpression reduces HDL-CE level in mice by increasing the clearance of HDL-CE [7]. Clinical trials have also revealed that APOF concentrations are lower in patients with hypertriglyceridemia than in healthy controls [8]. APOF is not only associated with lipid metabolism but also potentially with glucose metabolism and has recently been identified as a circulating biomarker associated with the risk of type 2 diabetes [9]. Given the established association between lipid metabolism and disease risk for various types of cancer, for example, prostate cancer and bladder cancer [10, 11], this is not difficult to understand that the expression level of APOF has been found to be helpful in colorectal cancer (CRC) and cholangiocarcinoma (CHOL) screening [12, 13], predicting prognosis in hepatocellular carcinoma (LIHC) [14], and HPV status in oropharyngeal squamous cell carcinoma in previous studies [15].

In this study, we drew the oncological data from the Cancer Genome Atlas (TCGA) to perform a pan-cancer analysis of APOF [16], primarily focusing on four types of cancer in which APOF is both differentially expressed between tumor and normal samples and prognostic-associated, including breast invasive carcinoma (BRCA), kidney chromophobe (KIRP), liver hepatocellular carcinoma (LIHC) and prostate adenocarcinoma (PRAD).

## Methods

### Differential and prognostic analysis

Similar to our previous studies [17, 18], we ﻿downloaded﻿ a standardized TCGA pan-cancer dataset from the UCSC database [19] and extracted the expression data of APOF in each sample. We also screened the metastatic samples from primary blood derived cancer-peripheral blood (TCGA-LAML), primary tumor and TCGA-SKCM. In addition, we obtained a high-quality TCGA prognostic datasets from the previous TCGA prognostic study [20]. We filtered the samples with the expression level of 0 and the samples with the follow-up time shorter than 30 days, and further performed log2 (*x* + 0.001) transformation for each expression value. We eliminated the cancer species with the number of samples less than 10, and finally obtained the expression data of 38 cancers and the data of overall survival (OS), cancer-specific survival (CSS), disease-free survival (DFS) and progression-free survival (PFS). Cox proportional hazards regression model was used to analyze the prognostic value of APOF on cancers, and log rank test was used to obtain prognostic significance. In terms of differential expression between tumor and normal samples, we screened the samples from sloid tissue normal, primary blood derived cancer-peripheral blood, primary tumor and removed the samples with the expression level of 0. Log2 (*x* + 0.001) transformation for each expression value was performed as well, and cancers with the number of samples less than 3 were removed. Finally, we obtained the expression data of 18 cancers, and unpaired Wilcoxon rank sum and signed rank tests were used to perform differential significance analysis. The clinical correlations of APOF in the pan cancer were evaluated as well. In this study, the abbreviations of each cancer from the TCGA database were shown in the Additional file 1: Fig. S1A.

### Tumor stemness, heterogeneity, and mutation landscape

Six tumor stemness indexes, namely differentially methylated probes-based (DMPss), DNA methylation based (DNAss), enhancer elements/DNA methylation-based (ENHss), epigenetically regulated RNA expression-based (EREG.EXPss), epigenetically regulated DNA methylation-based (EREG-METHss), RNA expression-based (RNAss) were used to analyze the correlation between stemness features and APOF expression through the Spearman analysis [21]. In addition, homologous recombination deficiency (HRD) [22], loss of heterozygosity (LOH) [22], neoantigen (NEO) [22], tumor ploidy [22], tumor purity [22], mutant-allele tumor heterogeneity (MATH) and tumor mutation burden (TMB) obtained from the GDC (https://portal.gdc.cancer.gov/) and proceeded by MuTect2 software and R package “maftools” [23], and microsatellite instability (MSI) [24] were used to assess the relationship between tumor heterogeneity and APOF expression. We integrated the mutation data and gene expression data, and we filtered the synonymous mutation samples. In each investigated cancer, we assessed the difference in the frequency of gene mutations between high- and low-expression of APOF according to the median expression of APOF through the chi-square test.

### RNA modifications and tumor immune microenvironment (TME)

We analyzed the correlations between APOF and 44 marker genes of three types of RNA modification (10 of m1A, 13 of m5C, and 21 of m6A) through the Spearman analysis. The correlations of 24 inhibitory and 36 stimulatory checkpoints [22], and 150 immunoregulatory genes (chemokine, receptor, MHC, immunoinhibitory, immunostimulatory [68]) with the mRNA expression of APOF were conducted as well. Timer [25] and ESTIMATE [26] algorithms were used to assess the TME using the R package “IOBR” [27]. In addition, we also analyzed the relationship between DNA methylation and mRNA expression of APOF, and the correlation of DNA methylation and mRNA expression of APOF with tumor infiltrating lymphocytes (TILs) were performed through the TISIDB database [28].

### Statistical analysis

We conducted all analyses through software R (version 3.6.3) and its suitable packages. Unpaired Wilcoxon rank sum and signed rank tests were used to analyze pairwise differences, and Kruskal test was used to test multiple sets of samples. Statistical significance was set as two-sided *p* < 0.05. Significance was marked as follows: *, *p* < 0.05; **, *p* < 0.01; ***, *p* < 0.001.

## Results

### Differential and prognostic analysis

**Fig. 1:**
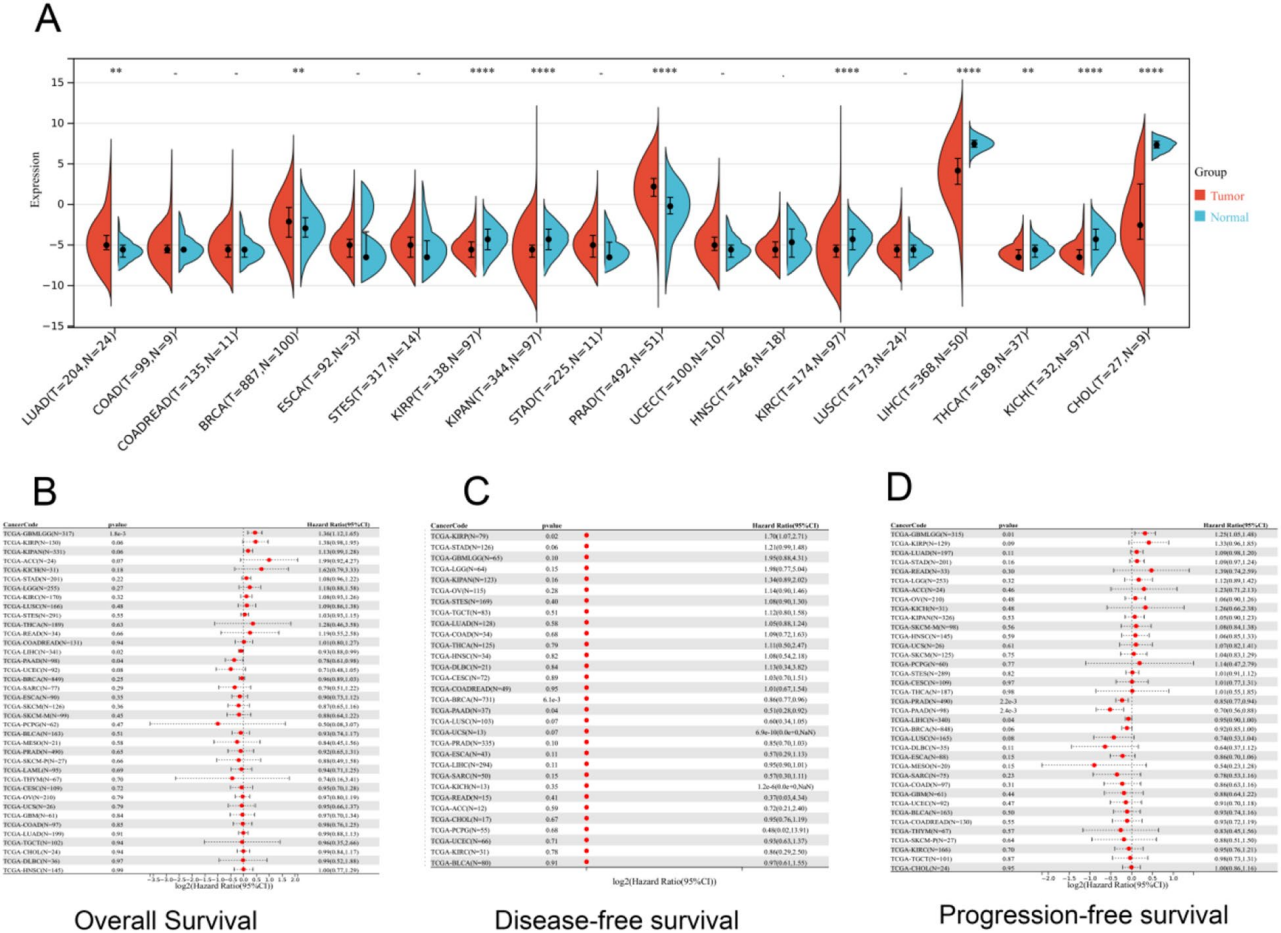
Differential expression and prognosis analysis of APOF. **A** Pan-cancer analysis of APOF for differential expression between tumor and normal tissues; **B** pan-cancer analysis of APOF for OS; **C** pan-cancer analysis of APOF for DFS; **D** pan-cancer analysis of APOF for PFS. OS: overall survival; DFS: disease-free survival; PFS: progression-free survival

Compared to normal samples, we observed that the APOF mRNA expression was significantly upregulated in lung adenocarcinoma (LUAD), low-grade glioma (LGG), PRAD and BRCA while downregulated in kidney renal papillary cell carcinoma (KIRP), pan-kidney cohort (KIPAN), kidney renal clear cell carcinoma (KIRC), LIHC, thyroid carcinoma (THCA), KICH and cholangiocarcinoma (CHOL) patients (Fig. 1A). In terms of OS, we found that high-expression APOF was significantly associated with poor prognosis in glioma (GBMLGG), and low-expression APOF was significantly associated with poor prognosis in LIHC and pancreatic adenocarcinoma (PAAD) (Fig. 1B). For CSS, we observed that overexpression of APOF was significantly related to GBMLGG and downregulation of APOF was significantly related to PAAD (Additional file 1: Fig. S1B). For DFS, high-expression APOF was significantly associated with poor prognosis in KIRP, and low-expression APOF was significantly associated with poor prognosis in BRCA and PAAD (Fig. 1C). In terms of PFS, high-expression APOF was significantly associated with poor prognosis in GBMLGG, and low-expression APOF was significantly associated with poor prognosis for PRAD and LIHC (Fig. 1D). In contrast, APOF expression levels were found to correlate with age and gender for LIHC (Additional file 1: Fig. S1C, D). Differential expression of APOF was significant among clinical grade and stage of LIHC (Additional file 1: Fig. S1E, F) as well as T stage for LIHC, N stage for PRAD (Additional file 1: Fig. S1G–I). Overall, we found that the common cancers differentially expressed between tumor and normal samples and prognostic-associated were BRCA, PRAD, KIRP, and LIHC in terms of DFS and PFS. Moreover, we also found that APOF was differentially expressed at different ages, with positive correlations for BRCA and LIHC, and negative correlations for PRAD.

### Tumor stemness, heterogeneity, mutation landscape, RNA modifications and immune regulatory genes and immune checkpoint

**Fig. 2:**
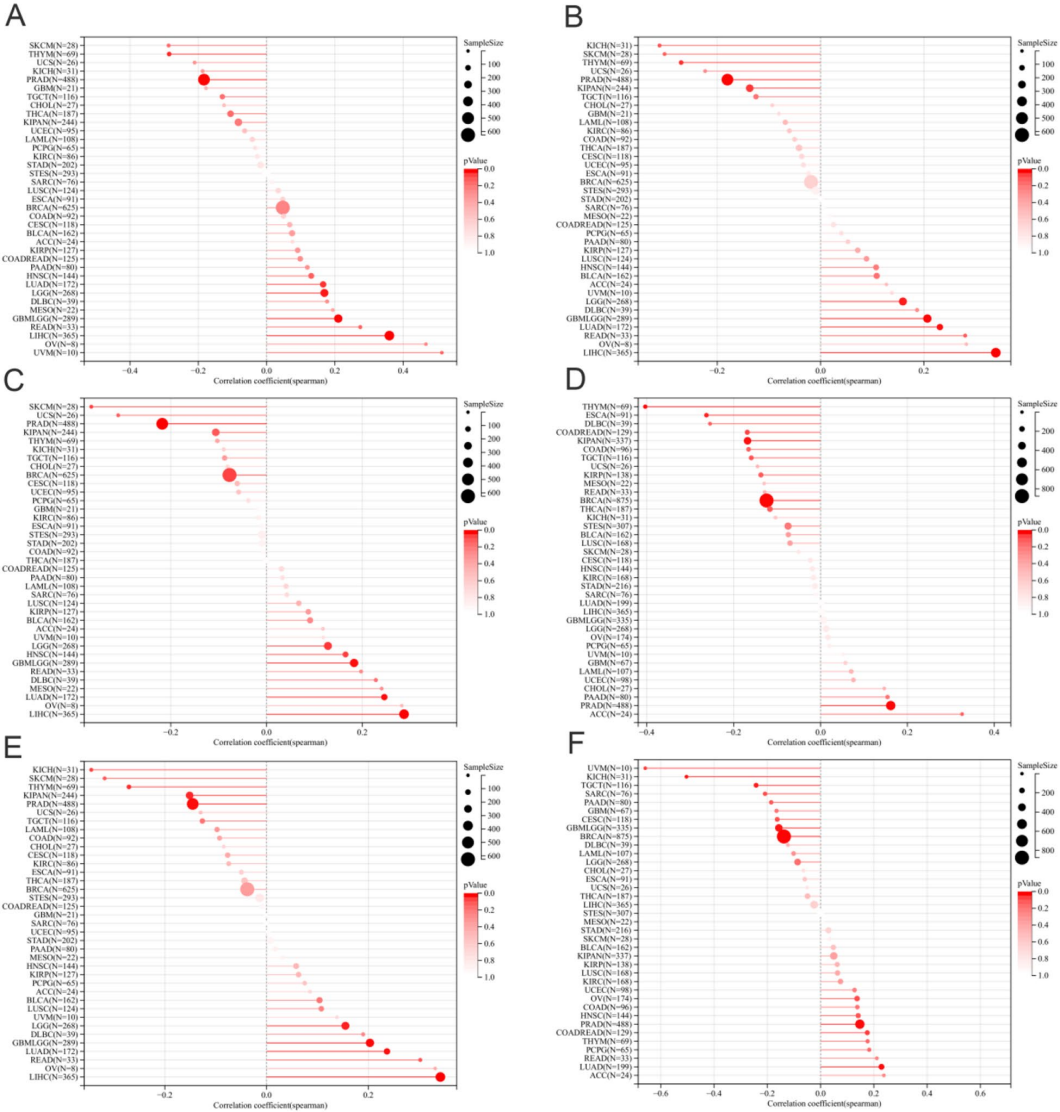
The pan-cancer Spearman analysis of tumor stemness and APOF expression. **A** The correlation between tumor stemness and APOF level using DMPss; **B** the correlation between tumor stemness and APOF level using DNAss; **C** the correlation between tumor stemness and APOF level using ENHss; **D** the correlation between tumor stemness and APOF level using EREG.EXPss; **E** the correlation between tumor stemness and APOF level using EREG-METHss; **F** the correlation between tumor stemness and APOF level using RNAss

**Fig. 3:**
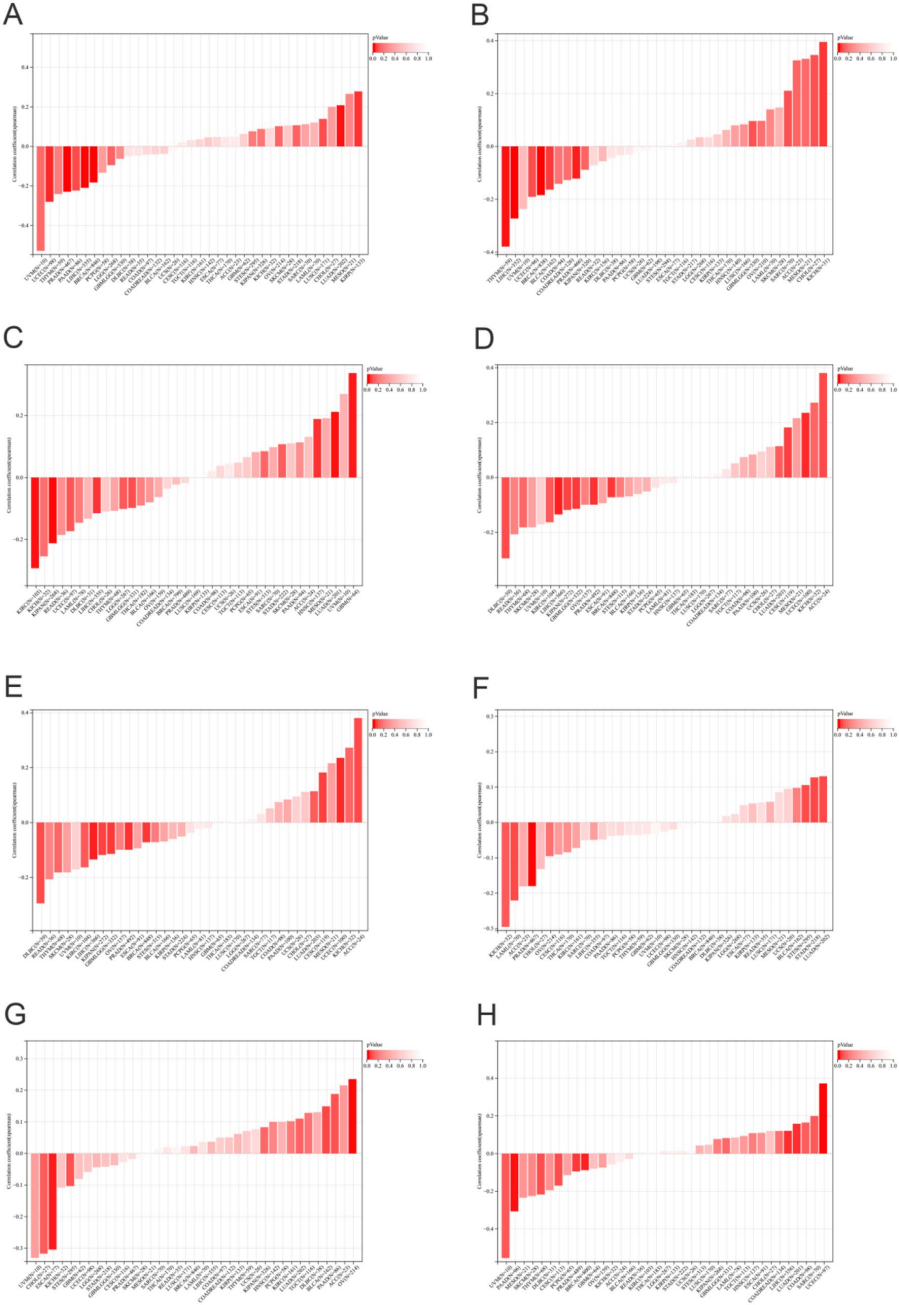
The pan-cancer Spearman analysis of tumor heterogeneity and APOF expression. **A** the correlation between HRD and APOF level; **B** the correlation between LOH and APOF level; **C** the correlation between MATH and APOF level; **D** the correlation between MSI and APOF level; **E** the correlation between NEO and APOF level; **F** the correlation between tumor ploidy and APOF level; **G** the correlation between tumor purity and APOF level; **H** the correlation between TMB and APOF level. HRD: homologous recombination deficiency; LOH: loss of heterozygosity; MATH: mutant-allele tumor heterogeneity; MSI: microsatellite instability; NEO: neoantigen; TMB: tumor mutation burden

The pan-cancer Spearman analysis showed that the mRNA expression of APOF was positively correlated with all six tumor stemness indexes (DNAss, EREG-METHss, DMPss, ENHss, RNAss and EREG.EXPss) with statistical significance for LUAD and with four tumor stemness indexes (DNAss, EREG-METHss, DMPss and ENHss) for LIHC (Fig. 2A–F). While APOF was negatively correlated with two tumor stemness indexes (RNAss and EREG.EXPss) for BRCA (Fig. 2A–F). In terms of tumor heterogeneity, for BRCA, the mRNA expression of APOF was negatively associated with TMB (*R* = − 0.09), MSI (*R* = − 0.07), NEO (*R* = − 0.07), HRD (*R* = − 0.18) and LOH (*R* = − 0.18) (Fig. 3A–H). For PRAD, the mRNA expression of APOF was negatively associated with TMB (*R* = − 0.09), MSI (*R* = − 0.10), NEO ((*R* = − 0.10), tumor ploidy (*R* = − 0.18), HRD (*R* = − 0.23) and LOH (*R* = − 0.12) (Fig. 3A–H). For KIRP, the mRNA expression of APOF was positively associated with HRD (*R* = 0.28) (Fig. 3A–H). For LIHC, the mRNA expression of APOF was positively associated with TMB (*R* = 0.12) while negatively associated with MATH (*R* = − 0.12), MSI (*R* = − 0.13), NEO (*R* = − 0.13), HRD (*R* = − 0.21) and LOH (*R* = − 0.27) (Fig. 3A–H).

**Fig. 4:**
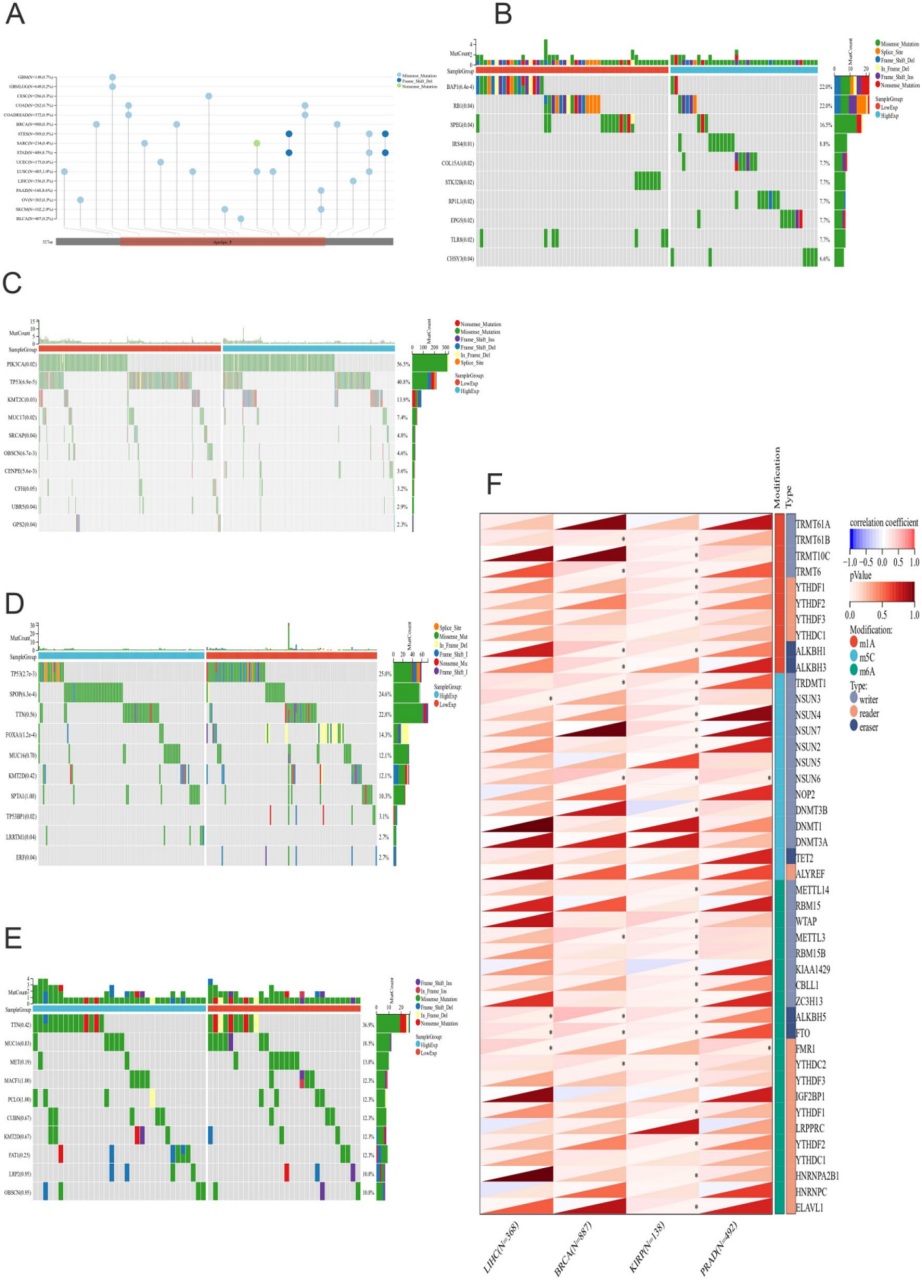
Mutation landscape of APOF and RNA modification. **A** Mutation landscapes of APOF for BRCA and LIHC; **B** the top 15 mutation genes between high and low-expression group for BRCA patients, including PIK3CA, TP53, KMT2C, MUC17, SRCAP, OBSCN, CENPE, UBR5, GPS2; **C** the top 15 mutation genes between high and low-expression group for PRAD patients, including TP53, SPOP, FOXA1, TP53BP1, LRRTM1 and ERF; **D** the top 15 mutation genes between high and low-expression group for KIRP patients, including TP53, SPOP, FOXA1, TP53BP1, LRRTM1 and ERF; **E** the top 15 mutation genes between high and low-expression group for LIHC patients. BAP1, RB1, SPEG, IRS4, COL15A1, STK32B, RP1L1, EPG5, TLR8 and CHSY3 mutations were significant; **F** the correlation of APOF expression and RNA modification genes

The mutation frequencies of BRCA and LIHC were 0.3% (Fig. 4A). We divided tumor patients into two groups according to the median expression of APOF. The mutations of PIK3CA, TP53, KMT2C, MUC17, SRCAP, OBSCN, CENPE, UBR5, GPS2 were significant between high- and low-expression group for BRCA (Fig. 4B). TP53, SPOP, FOXA1, TP53BP1, LRRTM1 and ERF mutations were significant for PRAD (Fig. 4C). In terms of KIRP, the genetic mutations were significant between high- and low-expression group, including TP53, SPOP, FOXA1, TP53BP1, LRRTM1 and ERF (Fig. 4C), and BAP1, RB1, SPEG, IRS4, COL15A1, STK32B, RP1L1, EPG5, TLR8 and CHSY3 mutations were significant for LIHC (Fig. 4E).

### RNA modifications and TME

**Fig. 5:**
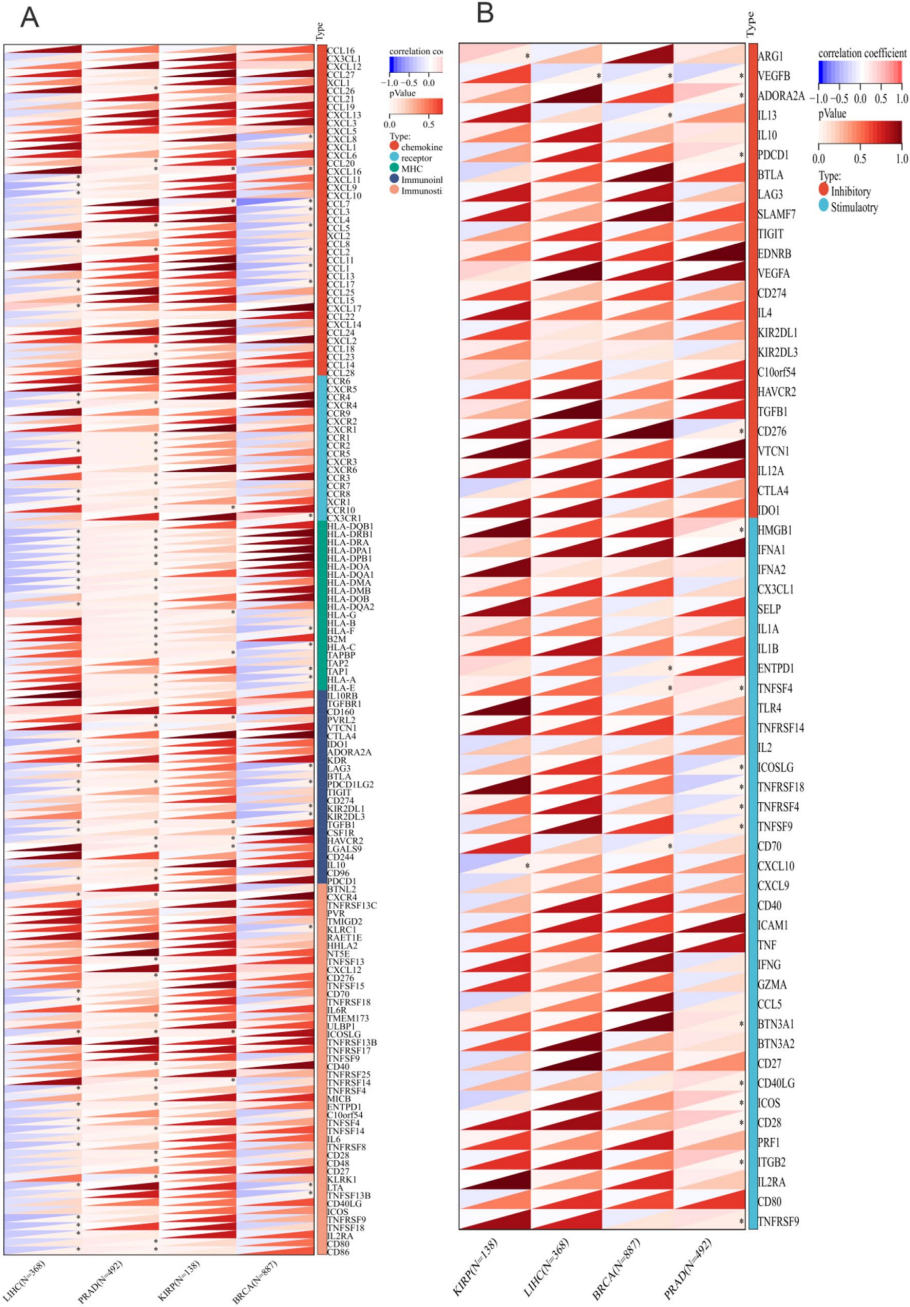
The Spearman analysis of APOF expression and immune checkpoints and regulatory genes. **A** The correlation of APOF expression with immune checkpoint genes; **B** the correlation of APOF expression with immune regulatory genes

In terms of RNA modifications, for PRAD, the mRNA expression of APOF was positively associated with NSUN6 and FMR1, while for BRCA, the mRNA expression of APOF was positively associated with TRMT61B, TRMT6, ALKBH1, ALKBH3, TRDMT1, NSUN6, METTL3, ALKBH5, FTO and YTHDC2 (Fig. 4F). For KIRP, multiple m1A, m5C and M6A modifications were found to be positively associated with APOF expression, while DNMT3B and KIAA1429 were negatively associated (Fig. 4F). NSUN3, ALKBH5, FTO and FMR1 were positively associated with the mRNA expression of APOF (Fig. 4F). Multiple immunoregulatory genes (Fig. 5A) as well as immune checkpoint genes (Fig. 5B) were found to be associated with APOF expression levels in all four cancer types.

**Fig. 6:**
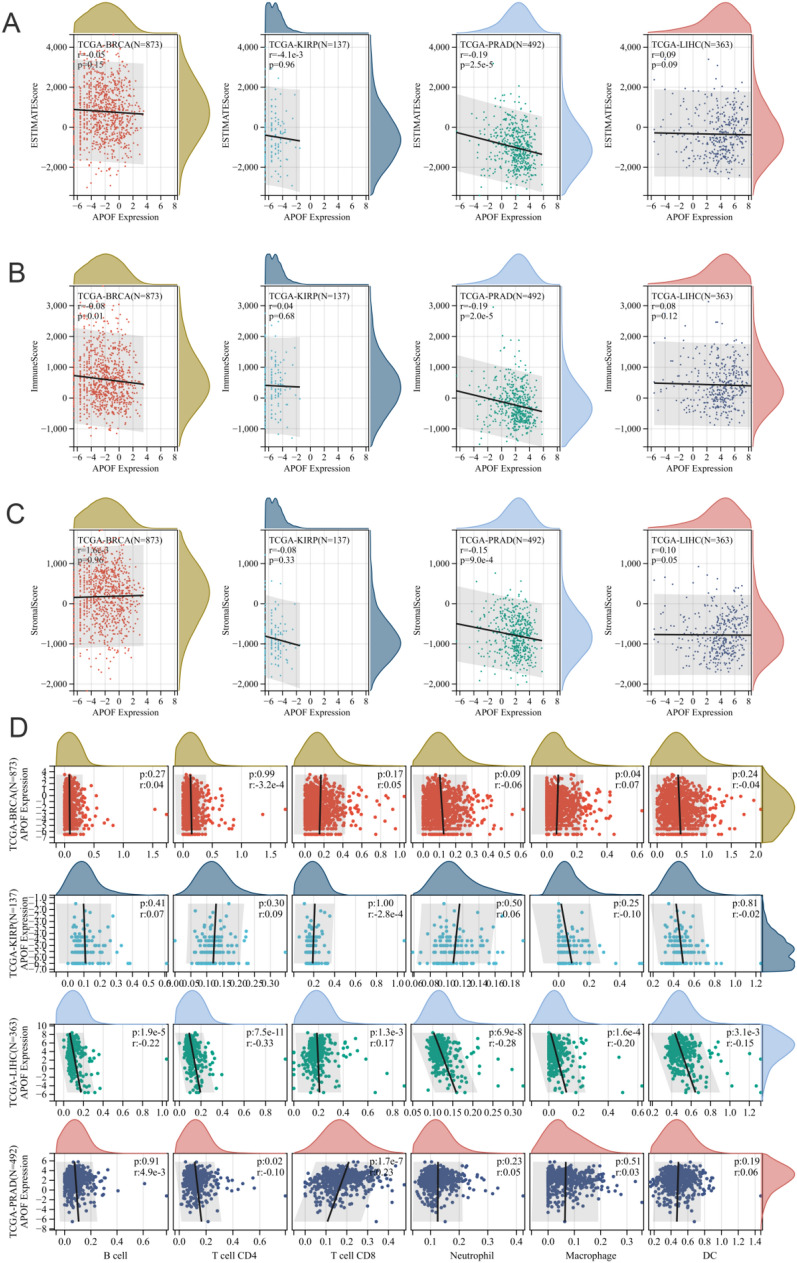
Tumor immune environment. **A** The correlation of APOF expression with ESTIMATE score; **B** the correlation of APOF expression with immune score; **C** the correlation of APOF expression with stromal score; **D** the correlation of APOF expression with immune infiltrating cells in the TIMER database

We found that for BRCA, APOF mRNA expression was negatively correlated with immune score (*R* = − 0.08) (Fig. 6B), while for LIHC, APOF mRNA expression was positively correlated with stromal score (*R* = 0.1) (Fig. 6C). For PRAD, APOF mRNA expression was negatively correlated with ESTIMATE score (*R* = − 0.19), immune score (*R* = − 0.19), and stromal score (*R* = − 0.15) (Fig. 6A–C). Meanwhile, we observed that the APOF mRNA expression was negatively associated with CD4+ T cells while positively associated with CD8+ T cells for BRCA patients (Fig. 6D). For LIHC, various TILs were negatively associated with APOF mRNA expression [29], including B cells, CD4+ T cells, neutrophils, macrophages and dendritic cells, while CD8+ T cells were positively associated (Fig. 6D).

## Discussion

Approximately 75% of circulating apolipoproteins are associated with high-density lipoproteins (HDL), with the remaining 25% present in LDL [30]. Apolipoprotein (APO) has been well studied in relation to cardiovascular illness, and its relationship with cancer has been gradually revealed [31, 32]. Other members of the APO family have been found to be involved in autophagy [33, 34], oxidative stress [35, 36], apoptosis [37], and cancer drug resistance [38, 39]. ApoF, a 162 amino acid C-terminal fragment of pro-APOF, is cleaved by PCSK7 to become mature with an apparent molecular weight of about 30 kDa [40]. ApoF is mainly synthesized and secreted by the liver and was found to be associated with HDL and to a lesser extent with LDL particles in circulation [1, 7, 29, 41]. APOF mainly functions by regulating CETP. Up to 70% of HDL cholesteryl ester clearance to the liver occurs through a CETP-dependent mechanism [42, 43]. Abnormal APOF expression may lead to abnormal lipid metabolism [30, 44]. Reprogramming of lipid metabolism is a known hallmark of cancer [45]. In tumor cells, the rate of lipogenesis is significantly accelerated. The higher rate of lipogenesis in cancer cells is intended to provide substances required for cell proliferation as well as energy generation through β-oxidation of fatty acids [46]. This metabolic reprogramming triggers a series of cascading events in tumor cell physiology and often produces harmful by-products such as ROS. APOF has also been implicated in immune and inflammatory responses in animal studies [47]. For example, transcription of interferon alpha (IFNα)-responsive genes was shown to be impaired in APOF knockout mice [32]. Our study found for the first time a correlation between APOF and the occurrence and prognosis of various cancers, particularly in the four types of cancer: BRCA, PRAD, KIRP, and LIHC. This relationship may be explained from the perspective of tumor metabolism and tumor stemness, heterogeneity, and immune infiltration.

It is known that cancer is closely related to age [48–51]. PRAD is one of the most common urinary tumors and its prevalence will be deteriorated as the population ages worldwide [48, 52–67]. BRCA and PRAD are two of the most important hormone-related tumors known. For tumorigenesis, since high CE was found to be positively associated with breast cancer, this is consistent with our findings that elevated APOF expression in tumors compared to normal tissue causes high cholesterol [68]. We proposed that APOF increases circulating cholesterol uptake by regulating cholesterol transport and esterification to meet the increased cholesterol demand of proliferating breast cancer cells. In contrast, for prognosis, the low APOF group had larger tumors, higher differentiation and proliferation rates, and more frequently occurring HER2-like phenotypes due to elevated LDL-CE, which further suggesting an important role for APOF in BRCA, by regulating lipid levels [69, 70]. In addition, the effect of hypercholesterolemia on mammary tumor growth and metastasis was also studied in APOE knockout mice [71]. Overall, targeting APOF, i.e., targeting cholesterol transport and esterification, may be one of the targets for BRCA, but might not be as effective as directly targeting downstream cholesterol uptake and its conversion with high specificity. The role of APOF in the carcinogenesis of BRCA patients is controversial, nevertheless. Lower levels of estrogen 2 (E2) can boost ETS-1 production and rapidly induced capillary angiogenesis in BRCA patients [72]. Meanwhile, ETS-1 can activate the APOF promoter [73].

Similar to BRCA, elevated APOF is observed during tumorigenesis, which leads to high cholesterol level and has been found to be positively associated with PRAD and the risk of developing aggressive PRAD [68, 70]. The white adipose tissue around the prostate is a source of lipids used by adjacent prostate cancer cells and a local factor that stimulates the progression of PRAD, where lipids can remodel extracellular matrix and support neovascularization [74]. In addition, hypercholesterolemia is associated with elevated androgen levels as well as the androgen receptor (AR) [75–78]. Whereas AR signaling may instead affect cholesterol synthesis. For example, androgen-responsive elements can upregulate the enzyme 3β-hydroxysterol Δ24-reductase (DHCR24) in AR-positive prostate cancer cells, thereby promoting cholesterol accumulation [78, 79]. In addition, our study suggested that APOF expression level was correlated with all prognostic indicators including OS, CSS, DFS and PFS in PAAD patients, although no differences were found between PAAD and normal tissues. Interestingly, we found that PRAD was the only cancer type in which APOF expression levels were negatively correlated with age, combined with our finding that APOF expression levels were much higher in PRAD tumor tissues than in normal tissues, due to the fact that PRAD is the only cancer type in which aging has been identified as a direct risk factor among all cancer types [80]. In addition, PAAD is also a hormone-related cancer and aggressive PAAD was found to be strongly upregulated on LDL-receptors in conjunction with increased cholesterol uptake [81]. Therefore, a prospective combined metabolic therapeutic strategy, in association with  other therapies, is a promising combined metabolic treatment option for PAAD [82].

The liver plays a key role in the metabolism of plasma apolipoproteins, and plasma lipid profiles may be altered in LIHC because plasma levels of apolipoproteins may be a sensitive marker of liver injury [83, 84]. Northern blot analysis showed that APOF mRNA was only found in liver tissue [4, 85]. Our research supported prior research on liver cancer cell lines that APOF expression is down-regulated in LIHC and is associated with low recurrence-free survival [14]. Our study additionally found that APOF was also associated with clinical stage and OS of LIHC patients. Since APOF expression inhibited the proliferation of LIHC cells in vitro and migrated slowly after APOF expression was upregulated [14]. Therefore, we hypothesize that APOF may play a role similar to that of a tumor suppressor gene and the one of the mechanisms of APOF-LIHC association is mediated through the intermediary of nonalcoholic fatty liver disease (NAFLD), which is a precancerous lesion of LIHC and can proceed to cirrhosis through fibrotic phase and can be exacerbated by LIHC [86]. APOF expression levels were reduced in mice on an obesogenic diet, which led to subsequent development of NAFLD and LIHC [87]. In addition, we found a large number of differences in the levels of TILs between the high and low groups of APOF mRNA expression levels, which were negatively correlated with all immune cells except CD8+ T cells. We postulate that the poor prognosis in patients with low APOF expression is associated with remodeling of the hepatic immune cell pool during NAFLD and involvement in the uncontrolled inflammatory environment that promotes liver injury and liver fibrosis [88]. In contrast, CD8+ T cells that can limit tumor load through their ability to initiate anti-tumor immune responses are instead observed to be reduced in the high-risk group [88, 89]. It is also interesting to note that our study found significant differences in APOF expression levels in male and female LIHC patients, with higher expression levels in men. In fact, in normolipidemic plasma, APOF level was 30% higher in men than in women and was positively associated with HDL and TG in normolipidemic men but not in women [8, 90]. This gender difference in APOF expression may be related to the role of HDL and TG in tumorigenesis. Care should be taken when APOF is used as a target of action.

KIRP, the incidence of which is much less studied than for KIRC, is the third most common type of RCC. Renal tumors are known to be characterized by high lipid content [91]. In our study, we found that unlike BRCA, PRAD and LIHC, APOF expression was elevated in normal tissues, and KIRP patients with high APOF expression had a worse prognosis, suggesting that APOF might not be a tumor suppressor gene for KIRP and has a tenuous relationship with immune checkpoints and immunomodulation. It is a question to be explored in the future.

Intuitively, diets rich in cholesterol or fatty acids would reduce liver APOF mRNA levels to less than half of the food-fed value [85]. However, little was previously known about the mechanisms regulating APOF gene expression. Shen et al. found that overexpression of C/EBPα and members of the ETS family increased APOF promoter activity in Huh7 cells, whereas knockdown of C/EBPα resulted in decreased APOF promoter activity in HepG2 and Huh7 cells [73]. FXR binds to and activates the FXR element ER1 in the promoter of the APOF gene [92]. For LIHC, C/EBPα is thought to activate the APOF promoter alone, from which it was hypothesized that mutations in the C/EBP binding site may almost completely eliminate APOF promoter activity [14]. Liu et al. found that APOF is also negatively regulated by agonist-activated LXR or PPARα nuclear receptors binding to a regulatory element ~ 1900 bases 5' to the APOF promoter [93].

Our research has certain limitations. Firstly, despite our observation that APOF was associated with various tumor types and tumor indicators, its specific mechanism cannot be presented through bioinformatics methods. On the other hand, we did not categorize patients of different races, according to ARIC study, there are significant racial disparities in lipid metabolism [10].

APOF is elevated during tumorigenesis in two hormone-dependent tumors, BRCA and PRAD, resulting in elevated circulating cholesterol levels by regulating cholesterol transport and esterification to supply the elevated cholesterol needs of tumor cells. Low APOF expression is then associated with poor prognosis for various tumor types, but the causal relationship might be the opposite of what we thought, and technical levels might in turn regulate a variety of lipid metabolic processes, including cholesterol accumulation. In the case of LIHC, it is most likely that the driving role of genes regulating lipid metabolism, including APOF, in NAFLD as well as in the process of LIHC is a direct result, and various immune cells in the NAFLD microenvironment could confirm this speculation. In contrast, for KIRP, APOF definitely regulates tumors in a different way. In the future, some approaches targeting APOF promoter regulation might be beneficial for the development of future therapeutic targets.

## Conclusions

Our pan-cancer study offered a relatively comprehensive understanding of the roles of APOF on BRCA, PRAD, KIRP, and LIHC.

## Supplementary Information

**Additional file 1: Figure S1.**the abbreviations of each cancer from the TCGA database;pan-cancer analysis of APOF for CSS;the correlation of APOF expression with age;the correlation of APOF expression with gender;the correlation of APOF expression with grade;the correlation of APOF expression with clinical stages;the correlation of APOF expression with T stages;the correlation of APOF expression with N stage;the correlation of APOF expression with M stages; CSS = cancer-specific survival.

## Data Availability

The datasets presented in this study can be found in online repositories. The names of the repository/repositories and accession number(s) can be found in the article/supplementary material.
