# Supplementary figures and images for: A pan-cancer analysis of the oncogenic and immunological roles of apolipoprotein F (APOF) in human cancer
Source: Eur J Med Res. 2023 Jun 14;28:190. doi: 10.1186/s40001-023-01156-w (PMC10265855; doi:10.1186/s40001-023-01156-w)

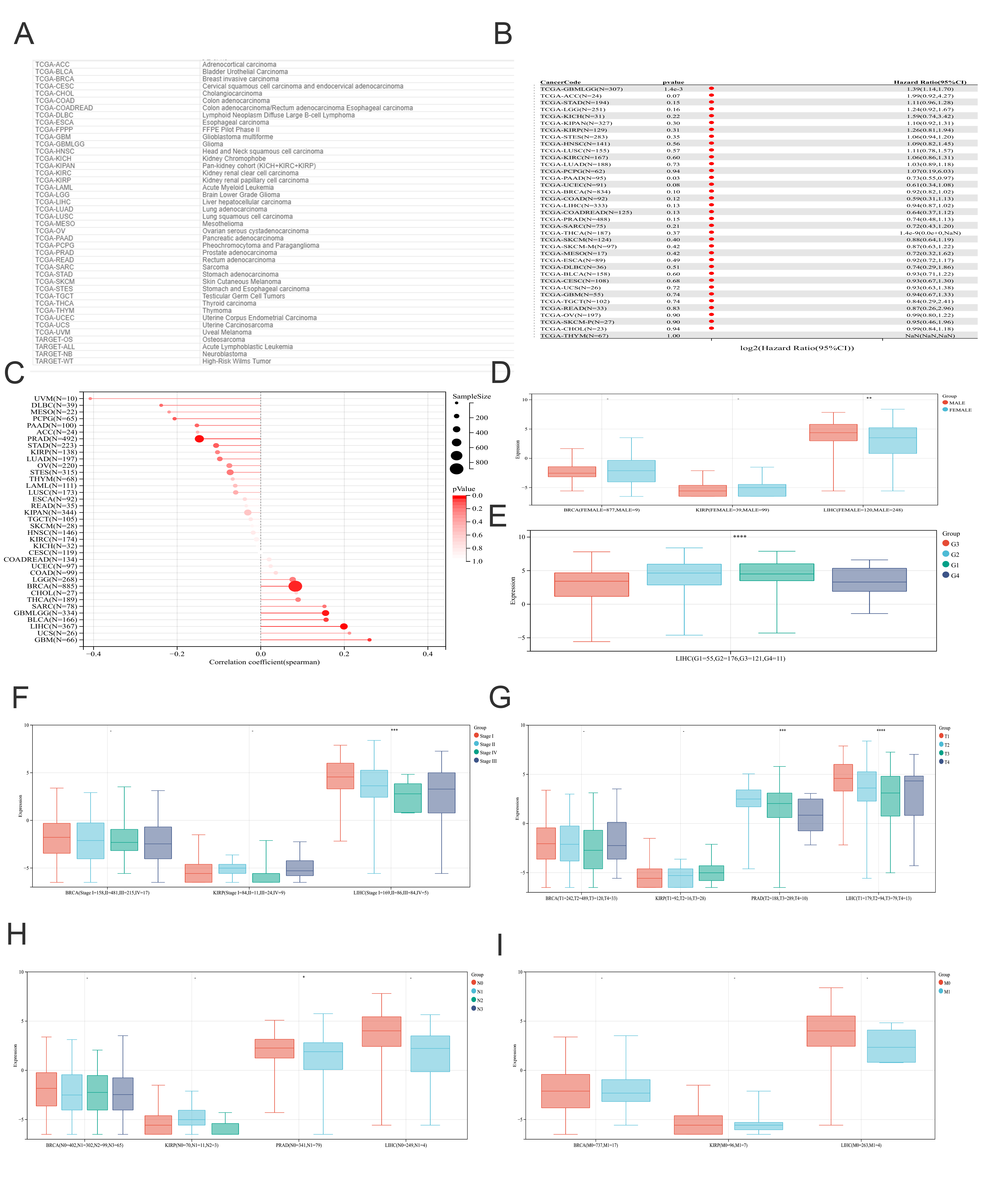

Supplement: Supplementary file 1 — Additional file 1: Figure S1.the abbreviations of each cancer from the TCGA database;pan-cancer analysis of APOF for CSS;the correlation of APOF expression with age;the correlation of APOF expression with gender;the correlation of APOF expression with grade;the correlation of APOF expression with clinical stages;the correlation of APOF expression with T stages;the correlation of APOF expression with N stage;the correlation of APOF expression with M stages; CSS = cancer-specific survival. [file 40001_2023_1156_MOESM1_ESM.png]
